# Cartilaginous predictors of residual acetabular dysplasia (RAD) in developmental dysplasia of the hip following closed or open reduction: A systematic review and meta-analysis

**DOI:** 10.3389/fped.2023.1124123

**Published:** 2023-03-29

**Authors:** Shuai Yang, Fei Su, Hao-Ruo Jia, Chen-Xin Liu, Qing-Da Lu, Ya-Ting Yang, Yong Liu, Jia-Ju Wang, Qiang Jie

**Affiliations:** ^1^ Pediatric Orthopaedic Hospital, Honghui Hospital, Xi'an Jiaotong University, Xi’an, China; ^2^ Medicle School of Yan'an University, Yan'an University, Yan’an, China

**Keywords:** developmental dysplasia of the hip, cartilage, acetabulum, prognosis, meta-analysis

## Abstract

**Object:**

This study was designed to analyze the cartilaginous predictors of residual acetabular dysplasia (RAD) after early treatment of developmental dysplasia of the hip and their diagnostic accuracy.

**Study design:**

Databases such as PubMed, Embase, Cochrane, and Web of science were searched to screen the literature. The quality of the literature was assessed by the QUADAS-2 tool. Qualitative and quantitative synthesis of literature were performed based on extracted data. For quantitative synthesis studies, the sensitivity, specificity, diagnostic odds ratio (DOR), and summary receiver operating characteristic (SROC) curve with corresponding confidence intervals were calculated.

**Results:**

For the cartilaginous acetabular index (CAI) group, the combined values of sensitivity, specificity, and DOR were 0.80 (95% CI = 0.54–0.93), 0.73 (95% CI = 0.57–0.84), and 10.62 (95% CI = 3.96–28.53), respectively. The corresponding values in the cartilaginous center-edge angle (CCE) group were 0.71 (95% CI = 0.57–0.82), 0.78 (95% CI = 0.66–0.87), and 8.64 (95% CI = 3.08–24.25), respectively. The area under the curve (AUC) of SROC was 0.82 (95% CI = 0.78–0.85) and 0.80 (95% CI = 0.76–0.83) for the CAI and CCE groups. The CAI group had higher sensitivity, DOR, and AUC than the CCE group.

**Conclusion:**

Both of these two groups have good diagnostic accuracy, and CAI/L-AI has a little edge over CCE/L-CEA. However, there is still more research needed to determine whether they can be used as independent indications for secondary orthopedic surgery.

**Systematic review registration:** [https://www.crd.york.ac.uk/PROSPERO/], identifier: [CRD42022338332].

## 1. Introduction

Developmental dysplasia of the hip (DDH) is a common musculoskeletal disorder in pediatric orthopedics, its prevalence in the population ranges from 0.1% to 3.4% (1). The pathological process includes hip dysplasia, subluxation, and complete dislocation (2). Osteoarthritis will develop more rapid and have to be treated by arthroplasty eventually if DDH is not identified timely and not treated in early childhood (3, 4). However, there is a substantial likelihood of residual acetabular dysplasia (RAD) even after systematic therapy in infancy and early childhood, and acetabuloplasty is needed to protect the hip in the later stage (5–8). There is still no uniform standard for the surgical timing and indications of RAD, so if some predictors can be identified to accurately assess and forecast the developmental prognosis after treatment, it can help to guide the timing of correction or avoid unnecessary surgery.

**Figure 1:**
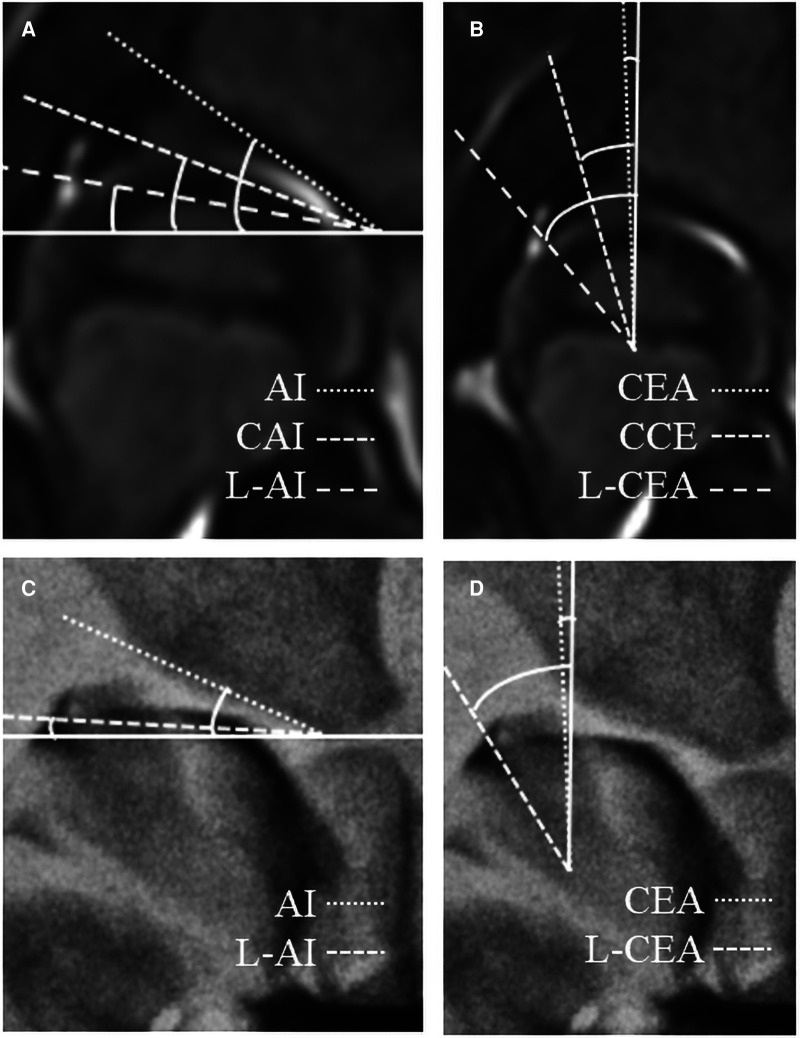
Some cartilaginous indicators measured on the coronal plane of MRI (**A,C**) and the anteroposterior film of hip arthrography (**B,D**). AI, acetabular index; CEA, centre-edge angle; CAI, cartilaginous acetabular index; CCE, cartilaginous center-edge angle; L, labral.

Previous researchers have proposed the use of acetabular index (AI), center edge angle (CEA), Reimer's index (RI), and center-head distance discrepancy (CHDD) as predictors of surgical indications (4, 9–16). However, all of these indices are measured in radiographs and only reflect bony acetabular development, not cartilaginous acetabular development, which represents the true potential of acetabular development (17–20). Therefore, some cartilaginous indicators based on MRI or hip arthrography have been proposed to be used as early warning indicators of RAD, such as cartilaginous acetabular index (CAI), cartilaginous centre-edge angle (CCE), labral acetabular index (L-AI) [It is also called Acetabular Cartilaginous Angle (ACA) by Zamzam et al. (20)], labral centre-edge angle (L-CEA) [It is also called the center-edge of the acetabular limbus angle (CEALA) by Satsuma et al.] (Figure 1). This meta-analysis summarized common cartilaginous predictors for assessing developmental prognosis after early treatment of DDH. And the most commonly used indicators by clinicians and researchers—CAI and L-AI (We define them as CAI group), CCE and L-CEA (We define them as CCE group), were combined separately to compare their diagnostic and prognostic performance.

## 2. Methods

This systematic review and meta-analysis was registered with PROSPERO (CRD42022338332) and was conducted based on the Preferred Reporting Items for Systematic Reviews and Meta-Analyses (PRISMA) guidelines (21).

### 2.1. Inclusion and exclusion criteria

The inclusion criteria follow: (1) The studies which used cartilaginous indicators to predict acetabular development after closed/open reduction in children with DDH; (2) The studies which reported sensitivity and specificity of predictors or 2 × 2 table data could be obtained by calculation.

The exclusion criteria follow: (1) Children with DDH who underwent osteotomy, juvenile adults or adults; (2) The femoral development rather than acetabular development, bone predictors rather than Cartilaginous ones; (3) Case reports or series, editorial notes, conference abstracts, abstract-only publications, opinion articles, animal trials, un-published studies were excluded; (4) Research sample size was less than 10.

### 2.2. Literature search strategy

The literature screened for this study was obtained from PubMed, Embase, Cochrane, and Web of science databases, with a time frame of database inception to June 2022. We used the following MeSH terms: “Developmental Dysplasia of the Hip”, “Cartilage”, “Acetabulum”, and “Prognosis”. Search strategies are shown in Supplementary Material.

### 2.3. Screening and literature selection

Two researchers independently screened the literature following strict inclusion and exclusion criteria. First, duplicate literature or duplicate data were eliminated. Additionally, an initial screening was performed with literature titles and abstracts. Finally, the remaining literature was further screened by reading the full text to determine the final included literature. If there were different opinions between the two researchers, the third researcher would assist in the determination to ensure the reliability of the included literature.

### 2.4. Data extraction

For qualitative synthetic articles, data extraction included: author, year of publication, country, and type of study; patient and hip sample size; methods of reduction, mean age at reduction; follow-up time; diagnostic index and reference standard; blind method; study results. For quantitative synthetic articles, additional extraction of true positive (TP), false positive (FP), true negative (TN), false negative (FN), sensitivity, specificity, cut-off value, and AUC were required.

### 2.5. Quality assessment

Our study used the Quality Assessment of Diagnostic Accuracy Studies (QUADAS-2 tool) (22) for the evaluation of the risk of bias and the clinical applicability of included studies. The QUADAS-2 tool consists of four important components: patient selection, index test, reference standard, flow and timing. Each of which is composed of several questions for a comprehensive assessment of the risk of bias (low, high, or unclear) (22). The quality assessment was performed by two investigators, and in case of disagreement, a third party assisted in the determination.

### 2.6. Statistical analysis

Risk bias evaluation of the included literature was performed using RevMan 5.4 software. Statistical analysis was performed using the MIDAS module of STATA 15.0, a bivariate mixed-effects model (23). Sensitivity, specificity, and diagnostic odds ratio (DOR) were analyzed, and *P *< 0.05 was considered statistically significant. A summary receiver operating characteristic (SROC) curve was also constructed to reveal the potential relationship between sensitivity and specificity (24). Fagan plot was developed to assess the clinical applicability of the index (25). Meta Disc 1.4 Spearman's correlation coefficient between the logarithm of sensitivity and the logarithm of (1-specificity) was calculated to analyze heterogeneity due to threshold effects. The Q-test and *I*^2^ index were used to evaluate the heterogeneity between studies (26), and the presence of moderate heterogeneity was implied when *P *< 0.05 for the *Q*-test and *I*^2^ ≥ 50% (26). Cook's distance was used as the sensitivity analysis's result to evaluate the stability of the study results. Deek's funnel plot asymmetry test was used to evaluate the publication bias of the included literature (26), and if *P *< 0.05, it suggested that publication bias exists.

## 3. Results

### 3.1. Literature search and study characteristics

**Figure 2:**
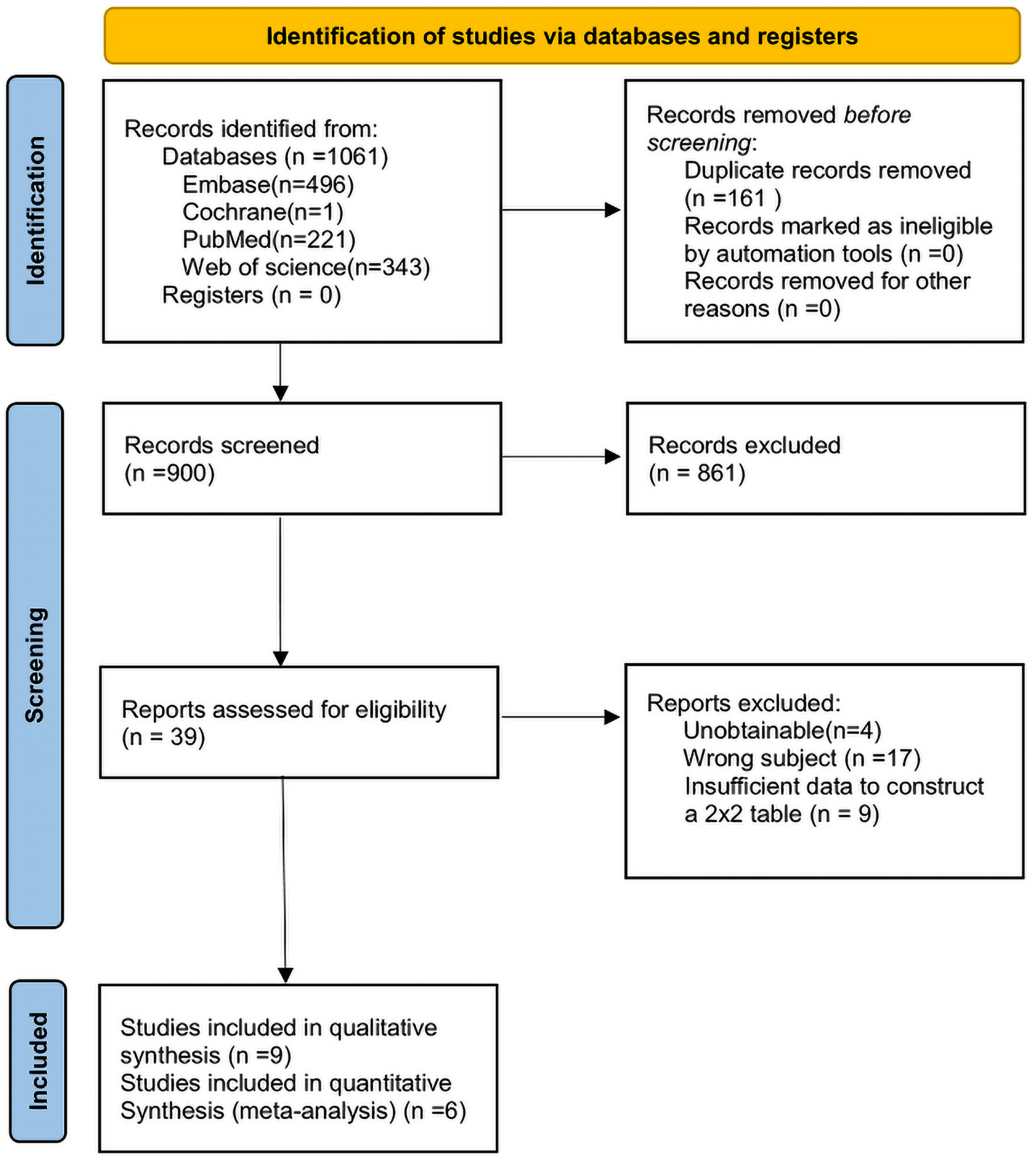
Flow-diagram of study screening.

Based on the search strategy of this study, a total of 1,061 relevant documents were retrieved from the database. We eliminated the duplicates and continued to exclude studies according to the title, abstract, inclusion, and exclusion criteria. There were 39 articles left for full-text reading and re-screening. And 4 of them were not available for full text, 17 studies did not match the subject, and 9 studies could not extract the 2 × 2 contingency table. Finally, 9 studies were included for qualitative synthesis (18, 27–34) and 6 studies for quantitative synthesis (18, 28, 29, 31, 33, 34). The search process and results are shown in Figure 2. The information on the characteristics of the 9 studies for qualitative synthesis is shown in Table 1. The information from the 6 quantitatively synthesized studies is shown in Table 2. Among them, there were 4 studies in the CAI group (18, 28, 31, 34) and 4 studies in the CCE group (18, 28, 29, 33).

**Table 1:** Characteristics of the studies included in qualitative synthesis.

Study, years	Trial type^a^	Country	Patient selection	Patients (n)	Hips (n)	Reduction method	Mean Age at Reduction (mon)	Diagnosis test^b^	Clinical reference standard test	Blinded	Mean age at final survey (y)	Findings/results
**Zhang 2021**	CC	China	Processed DDH	61	92	Closed/Open	‒	A-CAHI, L-CAHI, AHI, and A-CAHI combined L-CAHI	McKay standard	yes	5.4 ± 2.0	A-CAHI < 79. 1% or L-CAHI < 77. 3% indicates moderate or poor clinical function of the hip joint
**Tetsunaga 2021**	CC	Japan	Processed DDH	37	74	Open	22	L-AI, L-CEA	Severin classification	-	20	L-AI ≥4° and L-CEA <37° on MRI at 5 years of age need corrective surgery
**Takeuchi 2014**	CC	Japan	Suspected RAD	45	51	Pavlik harness/Closed/Open	8.4	CAI, CCE	Severin classification	-	11.7	CAI <18° or CCE >13° at 2 years of age show a good prognosis
**Shirai 2017**	CC	Japan	Suspected RAD	40	45	Pavlik harness/Closed/Open/Observation	7.2	MRI β angle	Severin classification	-	≥6	MRI β >65° at 3–4 years old indicate acetabular dysplasia
**Nakamura 2020**	CC	Japan	Processed DDH or LCPD^c^	92	92	Pavlik harness/Closed	‒	CCE	Severin classification	-	15.1	CCE ≥23° represents a good prognosis
**Satsuma 2016**	CC	Japan	Processed DDH	70	73	Pavlik harness/Closed	6	CEALA	The center-edge angle of Wiberg at age 18 years	-	18	For acetabular dysplasia with CEA from 0 to 10° at age 4, CEALA ≥45° indicate a poor prognosis
**Miyake 2018**	CC	Japan	Processed DDH	73	85	Open	17 ± 4.6	CAI	Severin classification	-	19 ± 5.7	CAI ≥ 10° at 5 years old may be used as an indicator of open reduction surgery
**Kawamura 2021**	CC	Japan	Processed DDH	37	74	Open	22	L-AR, L-CD	Severin classification	-	19	L-AR ≥ 23.1 or L-CD ≤ 8.5 on MRI at 5 years of age represents a good prognosis
**Johnson 2022**	CC	America	Processed DDH	48	63	Closed/Open	9.3 ± 3.2	CAI	the 90th percentile of normal boney AI values	yes	2 years after surgery	CAI >23° represents a poor prognosis at 2-year follow-up

^a^ RC, retrospective cohort; PC, prospective cohort; CC, case-control.

^b^ A-CAHI, anterior cartilaginous acetabulum-head-index; L-CAHI, lateral cartilaginous acetabulum-head-index; AHI, acetabulum-head-index; L-AI, labral acetabular index; L-CEA, labral center-edge angle; CAI, cartilaginous acetabular index; CCE, cartilaginous center-edge angle; CEALA, center-edge of acetabular limbus angle; L-AR, labral acetabular roof depth; L-CD, labral hip center distance.

^c^ LCPD, Legg-Calvé-Perthes disease.

**Table 2:** Characteristics of the studies included in quantitative synthesis.

Study, years	Diagnosis test	Imaging method	TP	FP	FN	TN	Sensitivity %	Specificity %	Cut-off value°	AUC
Johnson 2022	CAI	MRI	28	18	2	15	93	45	23	-
Miyake 2018	CAI	MRI	17	12	2	54	82	92	10	0.93
Takeuchi 2014	CAI	MRI	10	6	13	22	79	46	18	-
Tetsunaga 2021	L-AI	MRI	16	13	5	40	77	76	4	0.86
Nakamura 2020	CCE	MRI	11	10	1	70	92	88	23	0.93
Takeuchi 2014	CCE	MRI	15	8	8	20	72	64	13	-
Tetsunaga 2021	L-CEA	MRI	14	8	7	45	68	85	37	0.84
Satsuma 2016	CEALA	Arthrography	6	24	3	40	68	63	45	-

TP, true positives; FP, false positives; FN, false negatives; TN, true negatives.

### 3.2. Assessment of risk bias of included studies

**Figure 3:**
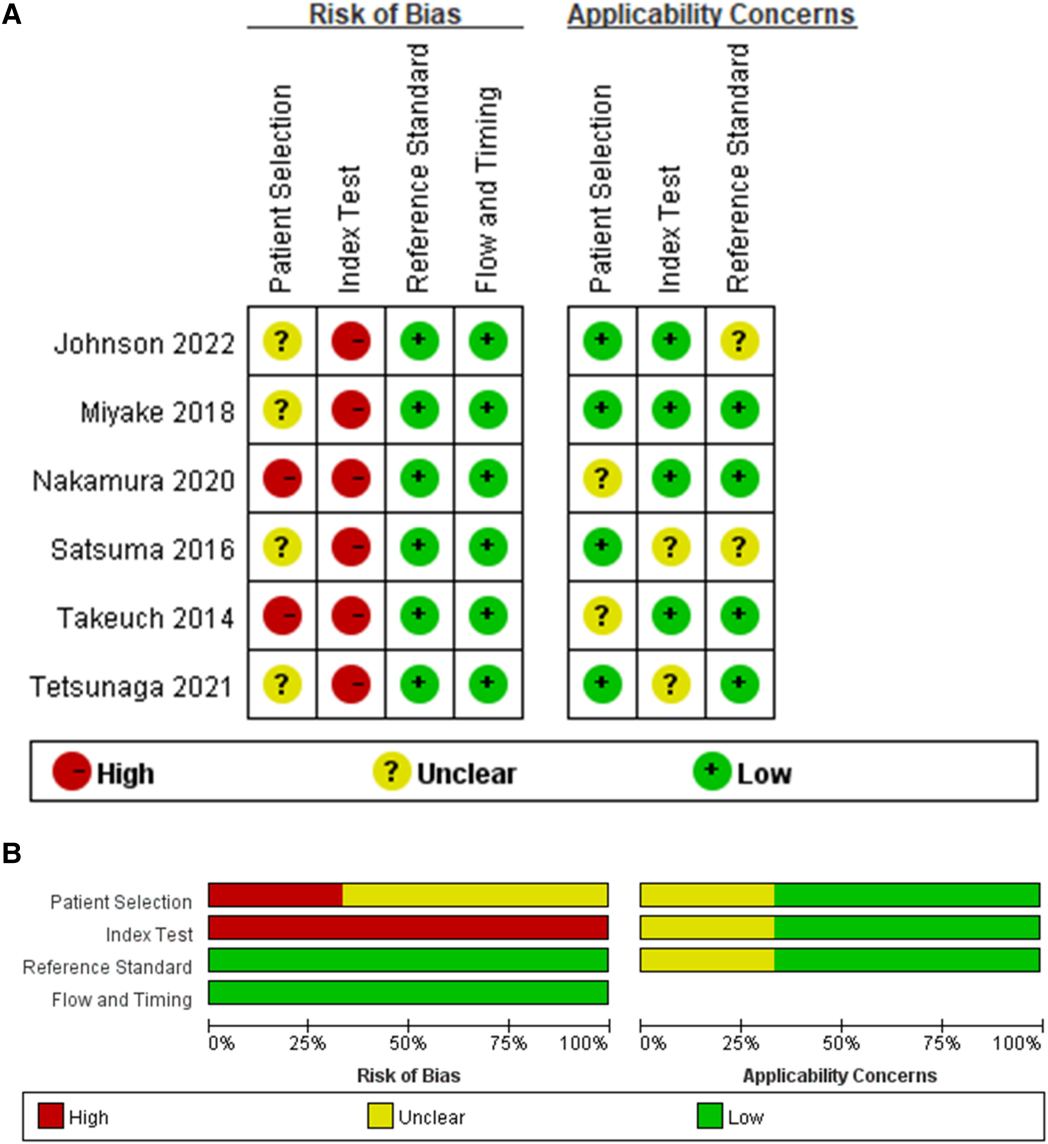
Quality assessment. (**A**) Risk of bias and applicability concerns graph. (**B**) Risk of bias and applicability concerns summary.

The quality of the included studies was evaluated by the QUADAS-2 tool (Figure 3), and the results showed that there are risk biases in “patient selection” and “index test”. In the “case selection”, 6 pieces of literature did not avoid case-control studies; in the “test to be evaluated”, the thresholds of all indicators were not predetermined but determined by the receiver operating characteristic (ROC) curve. However, the overall risk of bias in the 6 studies was within the acceptable range, and the clinical applicability evaluation was good.

### 3.3. Prognostic value of CAI group and CCE group in assessing acetabular development

**Figure 4:**
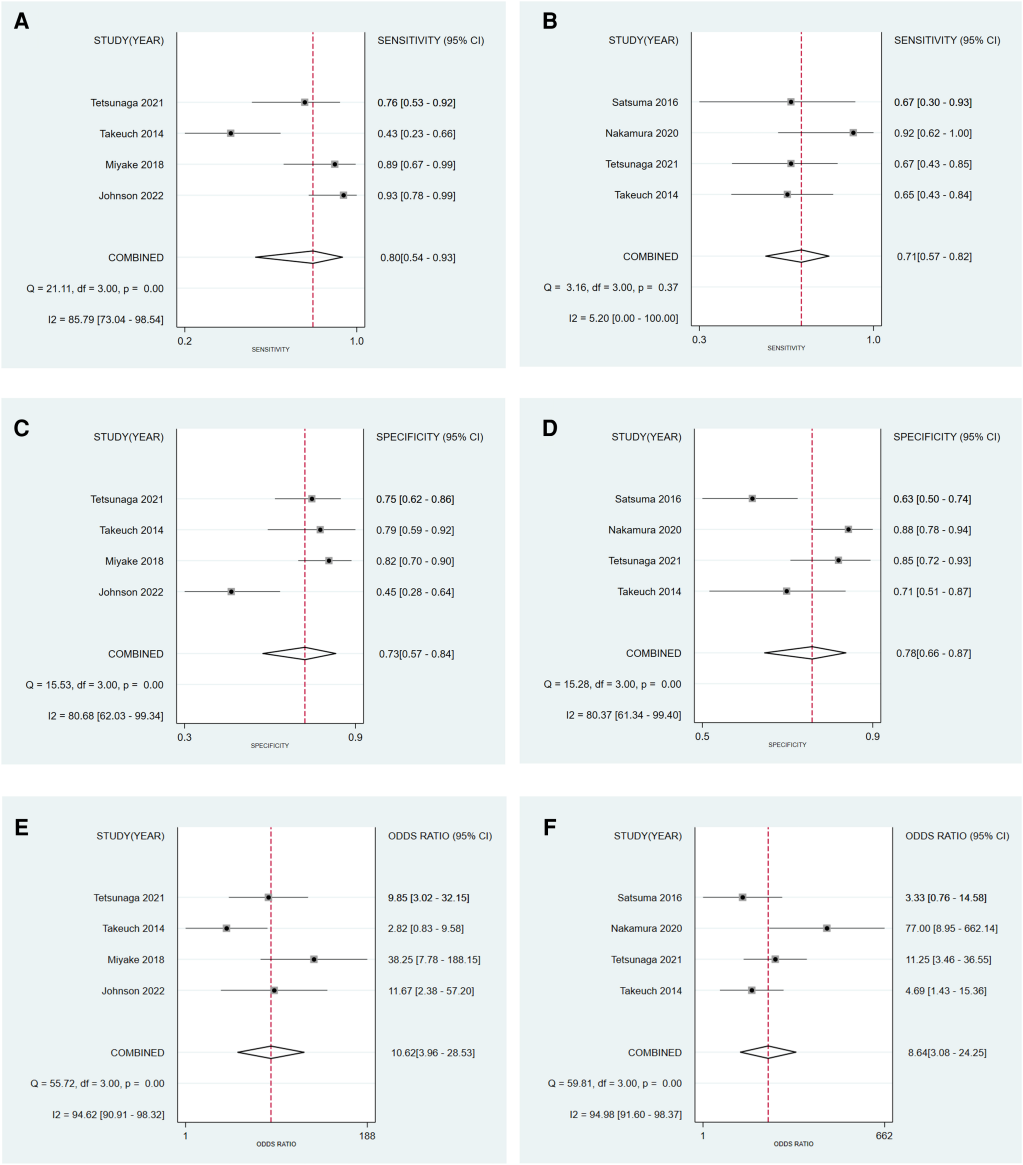
Forest plot to evaluate the sensitivity **(A)**, specificity **(C)**, and diagnostic odds ratio **(E)** of the CAI group, and the sensitivity **(B)**, specificity **(D)**, and diagnostic odds ratio **(F)** of the CCE group.

**Figure 5:**
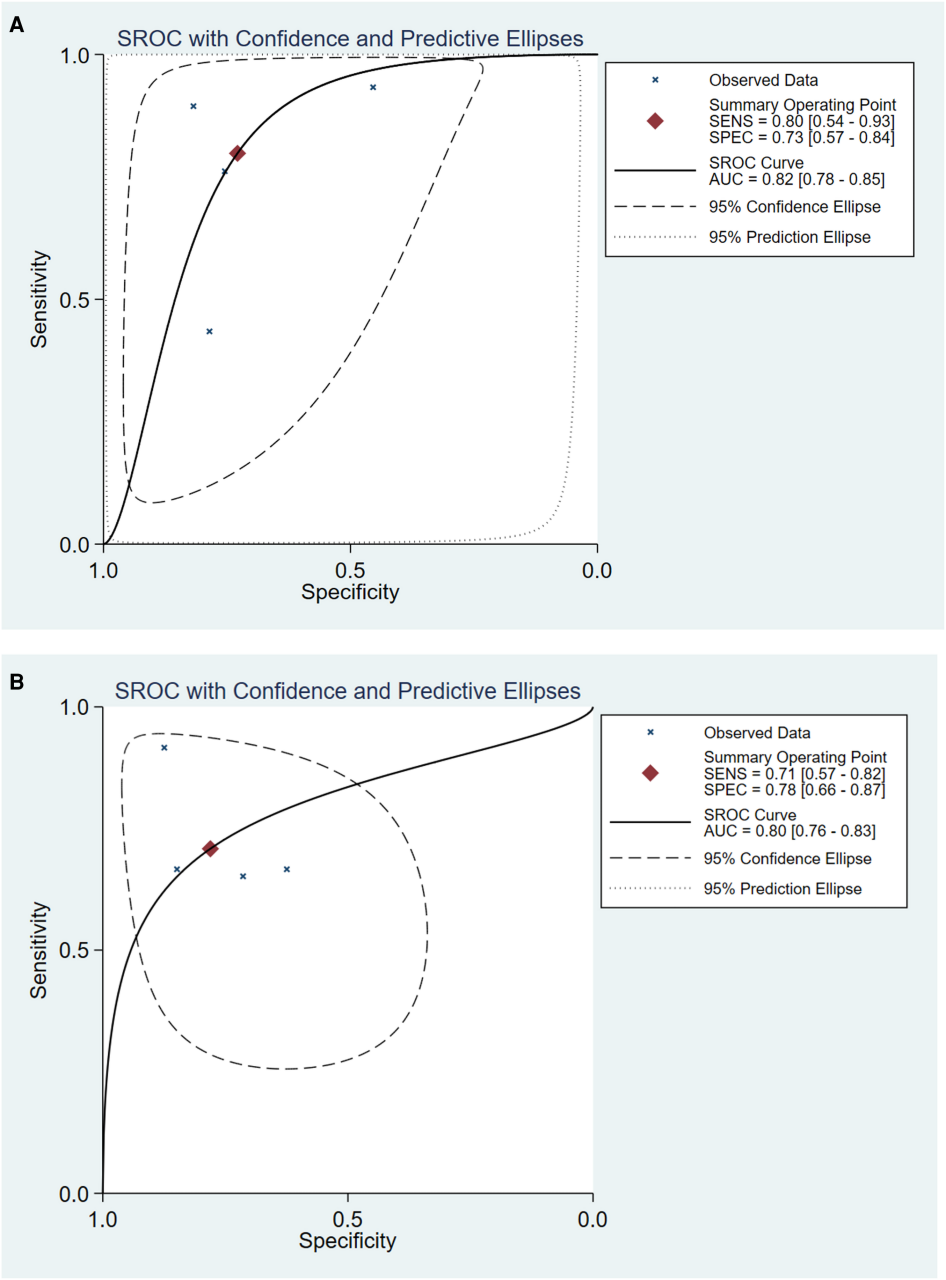
The SROC curve of CAI group (**A**) and CCE group (**B**) in evaluating acetabular development in DDH. SROC, summary receiver operator characteristic.

For the CAI group, pooled sensitivity = 0.80 (95% CI = 0.54–0.93), pooled specificity = 0.73 (95% CI = 0.57–0.84), pooled DOR = 10.62 (95% CI = 3.96–28.53) (Figures 4A–C). For CCE group, pooled sensitivity = 0.71 (95% CI = 0.57–0.82), pooled specificity = 0.78 (95% CI = 0.66–0.87), pooled DOR = 8.64 (95% CI = 3.08–24.25) (Figures 4D–F). Figure 5 showed the diagnostic and prognostic performance of the CAI group and CCE group on acetabular development based on SROC analysis. The results showed that the AUC of CAI was 0.82 (95% CI = 0.78–0.85), and the AUC of CCE was 0.80 (95% CI = 0.76–0.83). The value in the CAI group was higher than the CCE group in sensitivity, DOR, and AUC. Therefore, the CAI group has better prognostic performance than the CCE group.

### 3.4. Clinical application of CAI group and CCE group for assessing acetabular development

**Figure 6:**
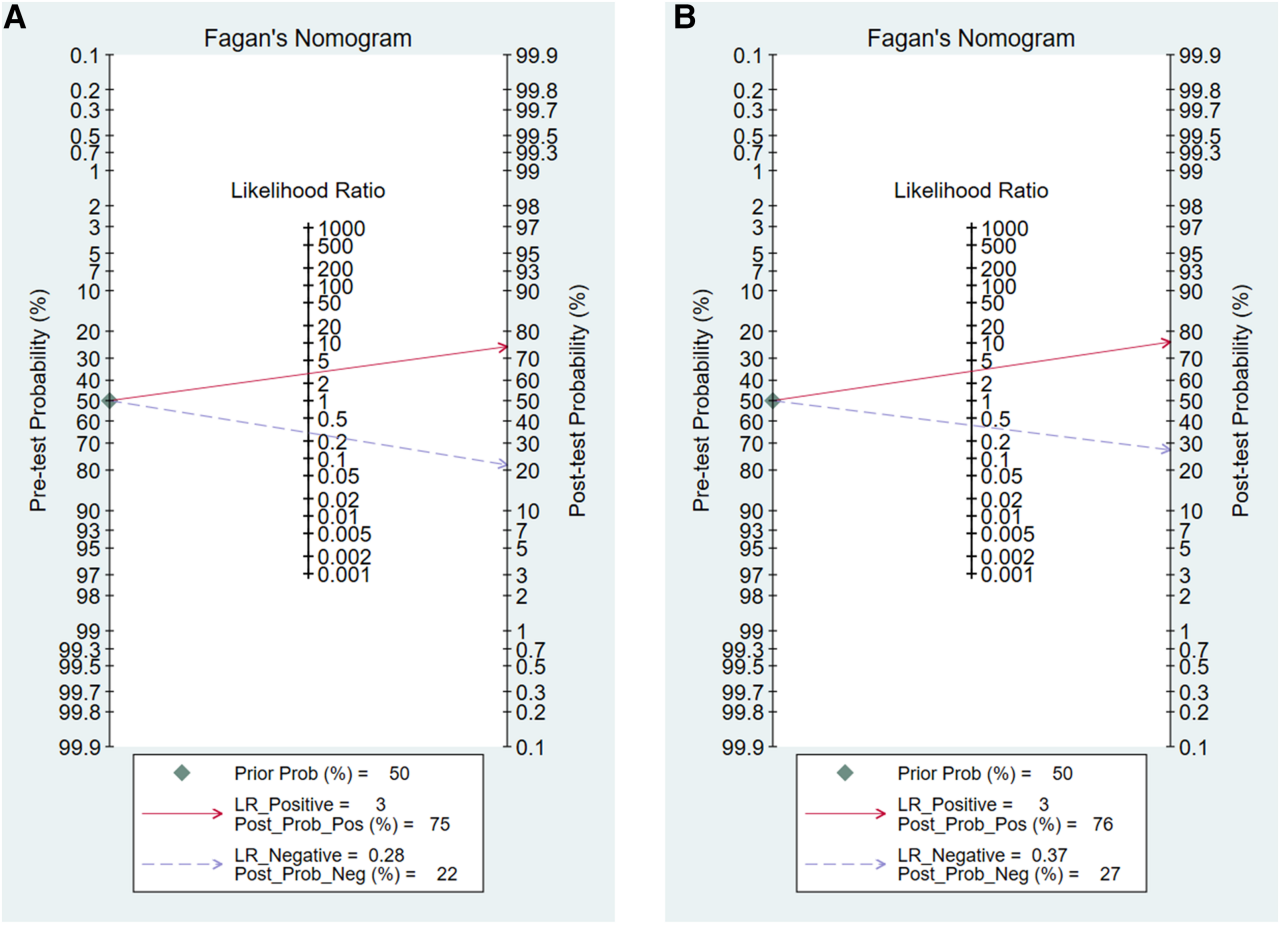
Fagan plot analysis to evaluate the clinical utility of CAI group (**A**) and CCE group (**B**).

Our study evaluated 50% pre-test probability and corresponding post-test probability. The Fagan plot analysis (Figure 6) showed that when the pre-test probability was 50%, the positive results in the CAI group predict a correct acetabular development at a probability of 75%, while 22% of negative results would be evaluated incorrectly. For the CCE group, the probability of a positive result correctly predicting acetabular development was 76% at a pretest probability of 50%, while 27% of patients with negative results would be evaluated incorrectly. It could be seen that the correct rate of positive results and the error rate of negative results in the CAI group were slightly lower than those in the CCE group.

### 3.5. Heterogeneity test of individual studies

Results of the heterogeneity test for the threshold effect were as follows: for the CAI group, the logarithm of sensitivity and logarithm of (1-specificity) were analyzed by Spearman correlation analysis, and the correlation coefficient was 0.400, *P* = 0.60 > 0.05, indicating that there was no heterogeneity caused by threshold effect. For the CCE group, Spearman correlation analysis between the logarithm of sensitivity and the logarithm of (1-specificity) showed a correlation coefficient of -0.632, *P* = 0.37 > 0.05, indicating that there was no heterogeneity caused by the threshold effect. The *I*^2^ test results of the CAI group showed that the sensitivity, specificity, and DOR were 85.79% (*P* < 0.05), 80.68% (*P* < 0.05), and 94.62% (*P* < 0.05), respectively, indicating the existence of heterogeneity caused by non-threshold effects (Figure 4). The *I*^2^ test results of the CCE group showed a sensitivity, specificity, and DOR of 5.20% (*P* = 0.37 > 0.05), 80.37% (*P* < 0.05), and 94.98% (*P* < 0.05), respectively, indicating the existence of heterogeneity caused by non-threshold effects. Considering the small amount of included studies, we did not conduct regression analysis and subgroup analysis to explore the source of heterogeneity.

### 3.6. Publication bias and sensitivity analysis

**Figure 7:**
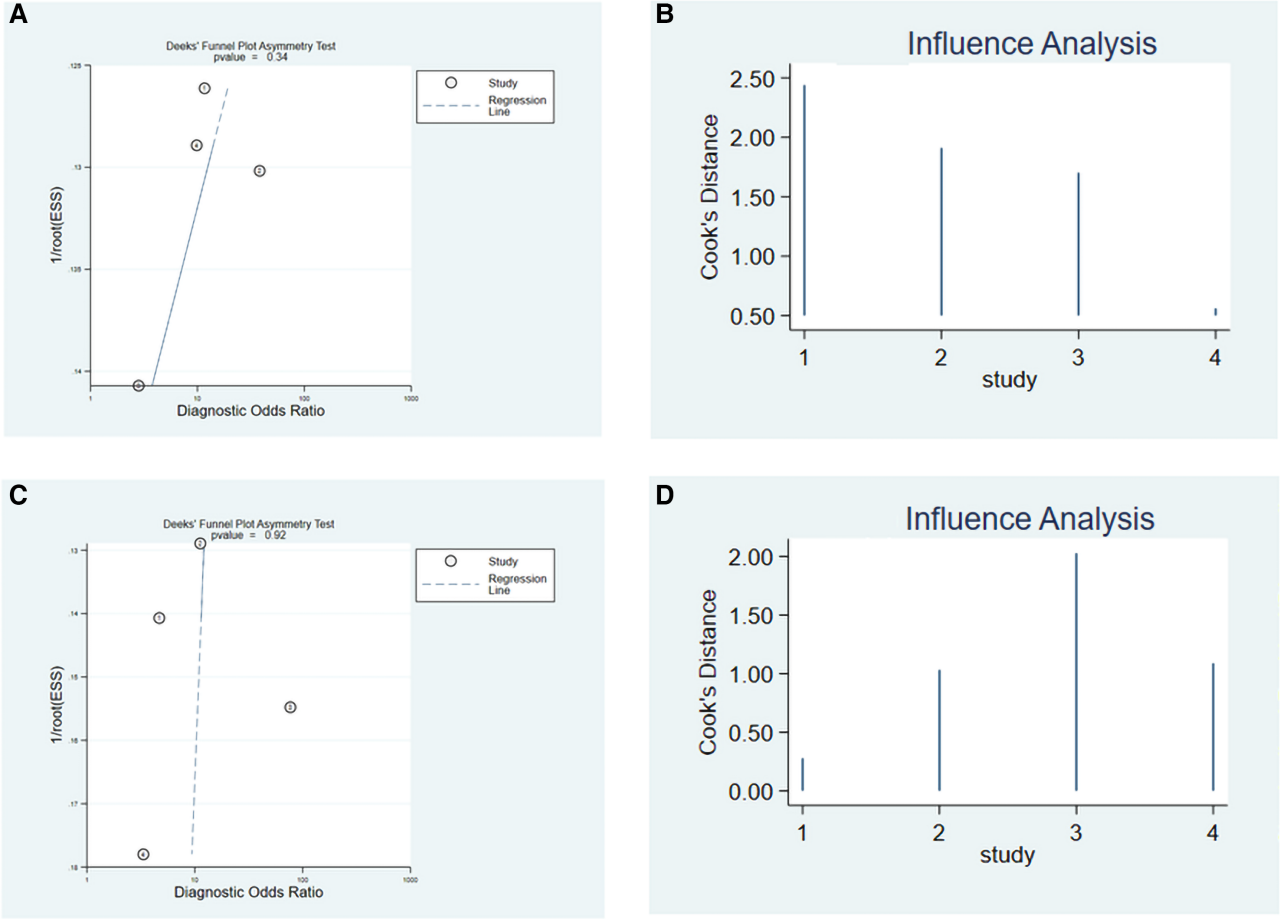
Deek's funnel plot asymmetry test to evaluate the publication bias of the CAI group **(A)** and CCE group **(C)**. Sensitivity analysis of CAI group **(B)** and CCE group **(D)** in predicting acetabular development.

Deek's test showed Bias = -1.25, *P* = 0.337 > 0.05 in the CAI group; and Bias = -0.11, *P* = 0.920 > 0.05 in the CCE group, suggesting that there was no significant publication bias in the included studies (Figures 7A,B). Sensitivity analysis showed that the original image made by STATA software does not mark any outliers, which means the studies included in the CAI group and the CCE group were relatively stable (Figures 7C,D).

## 4. Discussion

Even after successful early treatment, coverage of acetabulum on the femoral head may not be able to recover in children with DDH. Residual acetabular dysplasia (RAD) is a manifestation after early treatment of DDH and a common cause of secondary osteoarthritis (17, 35). David et al. (36) reported that 4% of children with early successful Pavlik harness treatment still had dysplasia. Malvitz and Weinstein (37) reported 54% of patients who underwent successful closed reduction remained dysplasia at long-term follow-up (mean, 30 years). Terjesen et al. (38) reported 38% of patients who underwent closed reduction remained dysplasia at skeletal maturity and 23% had undergone total hip replacement at a mean age of 43.7 years.

Previous scholars used bony indicators to evaluate the diagnosis of RAD and the determination of surgical indications (4, 9–16). However, many studies have shown that cartilage coverage represents the true potential of acetabular development (17–20). Therefore, using only bone indicators to evaluate RAD will lead to many unnecessary operations when acetabular coverage still had the potential for spontaneous improvement (39). Increasingly researchers are aware of this problem, so other indicators representing cartilage coverage have been proposed, and we have summarized these indicators in Table 1. It can be seen that research on cartilage indicators has emerged in recent years, and most of them have been carried out in Asia. All of the 9 studies included have adopted a case-control design. These studies basically selected children with DDH who had undergone closed or open reduction in the early stage for the follow-up and finally obtained the prediction results of a certain cartilage index for these patients. It is worth mentioning that most studies (6/9) selected Severin classification as the gold standard for judging the maturity of children, which is consistent with our meta-analysis because studies have confirmed the strong correlation between Severin classification and hip degenerative diseases (37, 38). Three studies did not select Severin classification as the gold standard, of which Satsuma 2016 and Johnson 2022 were included in our quantitative synthesis. The reference standards for these two studies were “the center edge angle of Wiberg at age 18 years” and “the 90th percentile of normal boney AI values”. Although we believe that both of them have the ability to distinguish between illness and health, we have marked their applicability to this study as “unclear” in the “Assessment of Risk Bias of included studies”, which has a minor impact on the overall applicability (Figure 3A). In addition, about the question “when does the potential for acetabular remodeling persist after early treatment”, the minimum age reported in the literature is 2 years old (40) or 2 years after treatment (15), and the maximum age is 11 years old (41). The follow-up time of all included studies exceeded the minimum acetabular remodeling time.

Six of the nine qualitative synthetic studies searched CAI, CCE, L-AI, and L-CEA, so we performed quantitative synthesis to compare who had better diagnostic and prognostic efficacy. There are two points worth noting. First of all, CAI and CCE can be measured by MRI or arthrography, which are two different imaging methods. Secondly, from the perspective of the anatomical structure of the acetabulum, some studies have only measured the angle of the cartilage part, that is, CAI and CCE (18, 29, 31, 34); other studies on the measurement of the angle include both the cartilage part and the labrum part, named L-AI and L-CEA (28, 33) (Figure 1). Although the two methods have different angles, they belong to the same type of measurement in principle, so we merge them into the same group and redefine them as the CAI group and CCE group. Different imaging modalities and different measurements may be important reasons for the heterogeneity caused by non-threshold effects. However, due to the small number of studies included in the two groups (both 4 studies), we did not perform subgroup analysis or regression analysis. Apart from the above two reasons, we found some other reasons that may cause heterogeneity: (1) different population distribution; (2) different gold standard selection. The specific effects of these factors will be revealed by adding more studies to a meta-analysis in the future.

Current studies have compared the prognostic performance of CAI and CCE grossly. Takeuchi et al. (18) found that CCE was more reliable than CAI in predicting the future development of acetabular. However, as two different measurement methods, there is no meta-analysis to explore which way has better diagnostic and prognostic efficacy. We innovatively combine the cartilage index (such as CAI) and the labrum index (such as L-AI) and then merge 6 original articles (503 patients) to compare the performance of the two measurement methods. From the results of this study, it can be seen that compared with the CCE group, the CAI group had higher sensitivity, DOR, and AUC in the prognosis assessment of DDH, indicating that the CAI group had better accuracy in RAD diagnosis and prompt.

The AUC of the two groups exceeded 0.80, indicating that both of them have good diagnostic accuracy. AUC values of 0.50 to 0.70, 0.70 to 0.90, and ≥0.90 represent low, moderate, and high diagnostic accuracy, respectively (42). As Onaç et al. (8) concluded that a series of indicators measured in MRI including CAI had a good correlation with persistent dysplasia, and these indicators could be used to predict the RAD of DDH patients after early treatment. However, when it came to specific clinical applications, Fagan plot analysis showed that in the case of a predicted probability of 50%, the CAI group and the CCE group had a 75% and 76% probability of correctly detecting positive results, which was, acetabular dysplasia. It can be seen that it is doubtful to use them as a single surgical indication, because it may cause overtreatment in more than 20% of cases. Therefore, for the prediction of RAD and the determination of surgical indications, it may be more accurate to use a combination of multiple indicators for a more comprehensive assessment.

This study also has some limitations. First of all, the studies included in the quantitative synthesis did not avoid the case-control design, and the threshold of the index was not predetermined but was calculated by the receiver operating characteristic (ROC) curve, which led to the low quality of the included studies in “patient selection” and “index test”. Secondly, the number of literature included in this meta-analysis is small, and we have not been able to perform subgroup analysis or meta-regression to explore possible sources of heterogeneity. Therefore, in the future, we need to include more studies to update this meta-analysis, and also expect more large cohort studies to reveal the clinical application of CAI and CCE in the prognosis of DDH.

In summary, the current research results provide some cartilage acetabular coverage indicators to predict RAD after early treatment, with CAI/L-AI and CCE/L-CEA most commonly used. Overall, these two groups have good diagnostic accuracy, the CAI group has a little edge over the CCE group in terms of accuracy. They serve as a valuable resource for acetabular development and the execution of subsequent orthopedic surgery, however, there is still more research needed to determine whether they can be used as independent indications for secondary orthopedic surgery.

## Data Availability

The original contributions presented in the study are included in the article/**Supplementary Material**, further inquiries can be directed to the corresponding authors.

## Supplementary material

SUPPLEMENTARY MATERIAL 1

Pubmed-search strategy.
