# Supplementary material for: Cartilaginous predictors of residual acetabular dysplasia (RAD) in developmental dysplasia of the hip following closed or open reduction: A systematic review and meta-analysis
Source: Front Pediatr. 2023 Mar 29;11:1124123. doi: 10.3389/fped.2023.1124123 (PMC10090413; doi:10.3389/fped.2023.1124123)
Supplement: Supplementary file 1 [file Datasheet1.pdf]

("Developmental Dysplasia of the Hip"[Mesh]) OR ((((((((Hip Dislocation, Developmental[Title/Abstract]) OR (Developmental Hip Dislocations[Title/Abstract])) OR (Dislocation, Developmental Hip[Title/Abstract])) OR (Developmental Hip Dislocation[Title/Abstract])) OR (Developmental Hip Dysplasia[Title/Abstract])) OR (Developmental Hip Dysplasias[Title/Abstract])) OR (Dysplasia, Developmental Hip[Title/Abstract])) OR (Hip Dysplasia, Developmental[Title/Abstract]))

AND

("Cartilage"[Mesh]) OR (Cartilages[Title/Abstract]) OR ("Acetabulum"[Mesh]) OR (((((((Acetabulums[Title/Abstract]) OR (Cotyloid Cavity[Title/Abstract])) OR (Cavities, Cotyloid[Title/Abstract])) OR (Cavity, Cotyloid[Title/Abstract])) OR (Cotyloid Cavities[Title/Abstract])) OR (Acetabula[Title/Abstract])) OR (Acetabulas[Title/Abstract]))

AND

(((((((prognosis[MeSH:noexp]) OR (diagnosed[Title/Abstract])) OR (cohort*[Title/Abstract])) OR (cohort effect[MeSH Terms])) OR (cohort studies[MeSH:noexp])) OR (predictor*[Title/Abstract])) OR(death[Title/Abstract])) OR (models, statistical[MeSH Terms])
